# Supplementary material for: Unveiling the Bioactive Potential of the Invasive Jellyfish Phyllorhiza punctata Through Integrative Transcriptomic and Proteomic Analyses
Source: Biomolecules. 2025 Aug 4;15(8):1121. doi: 10.3390/biom15081121 (PMC12383608; doi:10.3390/biom15081121)
Supplement: Supplementary file 1 [file biomolecules-15-01121-s001.zip › Suplementary Material/FileS3/PEPFOLD4/XP_065056523_12993/clusters.html]

 PEP-FOLD clusters


Download archive of all models  here   

|  |  |  |
| --- | --- | --- |
| # | file | sOPEP |
| # cluster 1 - Size: 1 |  |  |
|  | XP\_065056523\_12993-model1 | -8.1636 |
| # cluster 2 - Size: 7 |  |  |
|  | XP\_065056523\_12993-model2 | -6.99176 |
|  | XP\_065056523\_12993-model2.1 | -6.51308 |
|  | XP\_065056523\_12993-model2.2 | -5.26165 |
|  | XP\_065056523\_12993-model2.3 | -4.95452 |
|  | XP\_065056523\_12993-model2.4 | -4.32181 |
|  | XP\_065056523\_12993-model2.5 | -4.30623 |
|  | XP\_065056523\_12993-model2.6 | -3.56274 |
| # cluster 3 - Size: 7 |  |  |
|  | XP\_065056523\_12993-model3 | -6.83249 |
|  | XP\_065056523\_12993-model3.1 | -6.83249 |
|  | XP\_065056523\_12993-model3.2 | -5.83481 |
|  | XP\_065056523\_12993-model3.3 | -5.32497 |
|  | XP\_065056523\_12993-model3.4 | -3.97926 |
|  | XP\_065056523\_12993-model3.5 | -3.33555 |
|  | XP\_065056523\_12993-model3.6 | -3.31039 |
| # cluster 4 - Size: 3 |  |  |
|  | XP\_065056523\_12993-model4 | -6.39597 |
|  | XP\_065056523\_12993-model4.1 | -3.50549 |
|  | XP\_065056523\_12993-model4.2 | -3.09712 |
| # cluster 5 - Size: 2 |  |  |
|  | XP\_065056523\_12993-model5 | -5.19594 |
|  | XP\_065056523\_12993-model5.1 | -3.3189 |
| # cluster 6 - Size: 3 |  |  |
|  | XP\_065056523\_12993-model6 | -5.11975 |
|  | XP\_065056523\_12993-model6.1 | -4.62557 |
|  | XP\_065056523\_12993-model6.2 | -3.66797 |
| # cluster 7 - Size: 5 |  |  |
|  | XP\_065056523\_12993-model7 | -5.06976 |
|  | XP\_065056523\_12993-model7.1 | -4.29933 |
|  | XP\_065056523\_12993-model7.2 | -3.97337 |
|  | XP\_065056523\_12993-model7.3 | -3.54263 |
|  | XP\_065056523\_12993-model7.4 | -3.11383 |
| # cluster 8 - Size: 9 |  |  |
|  | XP\_065056523\_12993-model8 | -5.015 |
|  | XP\_065056523\_12993-model8.1 | -4.82929 |
|  | XP\_065056523\_12993-model8.2 | -4.70268 |
|  | XP\_065056523\_12993-model8.3 | -4.69476 |
|  | XP\_065056523\_12993-model8.4 | -4.46572 |
|  | XP\_065056523\_12993-model8.5 | -4.43551 |
|  | XP\_065056523\_12993-model8.6 | -4.28912 |
|  | XP\_065056523\_12993-model8.7 | -3.97352 |
|  | XP\_065056523\_12993-model8.8 | -3.67547 |
| # cluster 9 - Size: 5 |  |  |
|  | XP\_065056523\_12993-model9 | -4.97183 |
|  | XP\_065056523\_12993-model9.1 | -4.18983 |
|  | XP\_065056523\_12993-model9.2 | -4.1372 |
|  | XP\_065056523\_12993-model9.3 | -4.10397 |
|  | XP\_065056523\_12993-model9.4 | -3.16667 |
| # cluster 10 - Size: 4 |  |  |
|  | XP\_065056523\_12993-model10 | -4.89329 |
|  | XP\_065056523\_12993-model10.1 | -4.27119 |
|  | XP\_065056523\_12993-model10.2 | -4.08413 |
|  | XP\_065056523\_12993-model10.3 | -3.75329 |
| # cluster 11 - Size: 20 |  |  |
|  | XP\_065056523\_12993-model11 | -4.65156 |
|  | XP\_065056523\_12993-model11.1 | -4.3961 |
|  | XP\_065056523\_12993-model11.2 | -4.1215 |
|  | XP\_065056523\_12993-model11.3 | -4.09643 |
|  | XP\_065056523\_12993-model11.4 | -4.09489 |
|  | XP\_065056523\_12993-model11.5 | -3.91032 |
|  | XP\_065056523\_12993-model11.6 | -3.72843 |
|  | XP\_065056523\_12993-model11.7 | -3.56578 |
|  | XP\_065056523\_12993-model11.8 | -3.53669 |
|  | XP\_065056523\_12993-model11.9 | -3.53013 |
|  | XP\_065056523\_12993-model11.10 | -3.47732 |
|  | XP\_065056523\_12993-model11.11 | -3.44719 |
|  | XP\_065056523\_12993-model11.12 | -3.44242 |
|  | XP\_065056523\_12993-model11.13 | -3.42919 |
|  | XP\_065056523\_12993-model11.14 | -3.38014 |
|  | XP\_065056523\_12993-model11.15 | -3.36827 |
|  | XP\_065056523\_12993-model11.16 | -3.33835 |
|  | XP\_065056523\_12993-model11.17 | -3.33338 |
|  | XP\_065056523\_12993-model11.18 | -3.30001 |
|  | XP\_065056523\_12993-model11.19 | -3.29322 |
| # cluster 12 - Size: 5 |  |  |
|  | XP\_065056523\_12993-model12 | -4.24392 |
|  | XP\_065056523\_12993-model12.1 | -3.68053 |
|  | XP\_065056523\_12993-model12.2 | -3.45927 |
|  | XP\_065056523\_12993-model12.3 | -3.33207 |
|  | XP\_065056523\_12993-model12.4 | -3.30095 |
| # cluster 13 - Size: 1 |  |  |
|  | XP\_065056523\_12993-model13 | -4.1753 |
| # cluster 14 - Size: 1 |  |  |
|  | XP\_065056523\_12993-model14 | -4.07808 |
| # cluster 15 - Size: 3 |  |  |
|  | XP\_065056523\_12993-model15 | -3.89585 |
|  | XP\_065056523\_12993-model15.1 | -3.58963 |
|  | XP\_065056523\_12993-model15.2 | -3.02404 |
| # cluster 16 - Size: 4 |  |  |
|  | XP\_065056523\_12993-model16 | -3.79462 |
|  | XP\_065056523\_12993-model16.1 | -3.54234 |
|  | XP\_065056523\_12993-model16.2 | -3.40392 |
|  | XP\_065056523\_12993-model16.3 | -3.1915 |
| # cluster 17 - Size: 1 |  |  |
|  | XP\_065056523\_12993-model17 | -3.74085 |
| # cluster 18 - Size: 1 |  |  |
|  | XP\_065056523\_12993-model18 | -3.5851 |
| # cluster 19 - Size: 2 |  |  |
|  | XP\_065056523\_12993-model19 | -3.51154 |
|  | XP\_065056523\_12993-model19.1 | -3.18682 |
| # cluster 20 - Size: 3 |  |  |
|  | XP\_065056523\_12993-model20 | -3.43901 |
|  | XP\_065056523\_12993-model20.1 | -3.2429 |
|  | XP\_065056523\_12993-model20.2 | -3.16211 |
| # cluster 21 - Size: 5 |  |  |
|  | XP\_065056523\_12993-model21 | -3.43796 |
|  | XP\_065056523\_12993-model21.1 | -3.37932 |
|  | XP\_065056523\_12993-model21.2 | -3.34067 |
|  | XP\_065056523\_12993-model21.3 | -3.30954 |
|  | XP\_065056523\_12993-model21.4 | -3.24129 |
| # cluster 22 - Size: 2 |  |  |
|  | XP\_065056523\_12993-model22 | -3.43168 |
|  | XP\_065056523\_12993-model22.1 | -3.28901 |
| # cluster 23 - Size: 3 |  |  |
|  | XP\_065056523\_12993-model23 | -3.25677 |
|  | XP\_065056523\_12993-model23.1 | -3.22519 |
|  | XP\_065056523\_12993-model23.2 | -3.18905 |
| # cluster 24 - Size: 1 |  |  |
|  | XP\_065056523\_12993-model24 | -3.25671 |
| # cluster 25 - Size: 2 |  |  |
|  | XP\_065056523\_12993-model25 | -3.03805 |
|  | XP\_065056523\_12993-model25.1 | -3.00961 |
|  |  |  |
